# Apremilast as an Adjuvant in Refractory Reactive Arthritis: A Retrospective Observational Study

**DOI:** 10.7759/cureus.104968

**Published:** 2026-03-10

**Authors:** Noorussaba Arfeen, Shivangi Singh, Vikas Shankar, Kaushal Kishore

**Affiliations:** 1 Internal Medicine, Patna Medical College, Patna, IND; 2 Skin and VD, Patna Medical College, Patna, IND; 3 Skin and VD, Nalanda Medical College, Patna, IND

**Keywords:** acr, apremilast, phosphodiesterase 4 (pde4) inhibitor, : reactive arthritis, reiter syndrome

## Abstract

Background and objective

Reactive arthritis (ReA), formerly termed Reiter’s syndrome, is an autoimmune inflammatory arthritis that develops in response to a gastrointestinal or genitourinary infection. Although nearly two-thirds of patients have a self-limited course, the remaining patients go on to develop chronic symptoms and complications. In the absence of a curative treatment, the treatment goal remains the alleviation of symptoms, for which diverse treatments have been used with variable responses. Apremilast, an oral phosphodiesterase 4 (PDE4) inhibitor typically used for psoriatic arthritis and plaque psoriasis, appears to be a promising therapeutic option for ReA. This study aimed to evaluate the efficacy and safety of oral apremilast as an adjuvant therapy in patients with treatment-refractory ReA. In this study, adjuvant therapy refers to the addition of apremilast to ongoing conventional treatment with the aim of improving disease control and facilitating the reduction or discontinuation of concomitant medications such as corticosteroids and non-steroidal anti-inflammatory drugs (NSAIDs).

Material and methods

This was a retrospective, observational pilot study conducted at a tertiary care center. Medical records of 12 adult patients with treatment-refractory ReA, defined according to the criteria of the Third International Workshop on Reactive Arthritis (1996), who received oral apremilast as adjuvant therapy for 40 weeks between June 2024 and May 2025, were reviewed. Apremilast was administered at a dose of 30 mg twice daily following one week of dose titration. Treatment efficacy was primarily assessed using the American College of Rheumatology 20% improvement criteria (ACR20) along with secondary outcomes including ACR50/70 responses, changes in swollen and tender joint counts, inflammatory markers, improvement in functional outcomes measured by the Health Assessment Questionnaire-Disability Index (HAQ-DI) status, and evaluation of the corticosteroid-sparing effect.

Results

The mean age of the patients at the time of diagnosis was 26.91 years (range 19-36 years). Of the patients receiving apremilast (n=12), 10 (83.33%) achieved an ACR20 response at week 40. Although observed in a smaller proportion of patients, the higher-efficacy outcome measures, such as ACR50 and ACR70 responses, were sustained over 40 weeks in eight (66.67%) and five (41.67%) patients with continued treatment. The mean swollen joint count (SJC) and tender joint count (TJC) improved by 79.3% and 68.8%, respectively, at week 40. Around two-thirds of the treated patients (n=8, 66.67%) achieved a HAQ-DI minimal clinically important difference of ≥0.35 at 40 weeks. No serious infections or any other severe adverse effects were recorded in any of the patients.

Conclusions

Apremilast appears to be an efficacious and safe novel therapeutic option for treatment-refractory ReA. In addition to providing direct and substantial improvements in joint-related symptoms and beneficial effects on extra-articular manifestations, its corticosteroid-sparing effect makes it a promising candidate as an adjuvant in the treatment of ReA. The results of this study warrant further validation in larger controlled trials.

## Introduction

Reactive arthritis (ReA) generally develops several days to weeks after either a gastrointestinal infection caused particularly by species of the genera Salmonella, Shigella, Yersinia, Campylobacter, and Clostridium, or more commonly after a genitourinary infection typically caused by Chlamydia trachomatis, Neisseria gonorrhoeae, Mycoplasma hominis, and Ureaplasma urealyticum [1]. Approximately 10% of patients have never had a symptomatic infection. Histocompatibility leukocyte antigen B-27 (HLA-B27), a major histocompatibility complex (MHC) class I molecule involved in T-cell antigen presentation, is linked to ReA in roughly 75% of instances [2]. Patients with HLA-B27 positivity tend to suffer from more severe and long-term disease.

ReA is an immune-mediated syndrome in which inflammatory changes in the joints leading to arthritis are triggered by the persistent activation of host cytotoxic and helper T cells induced by bacterial fragments, such as lipopolysaccharide and nucleic acids [3]. Although the precise interaction between the inciting microbe and host immune cells that leads to the development of ReA remains to be elucidated, molecular mimicry-associated cross-reaction between microbial antigens and self-proteins, which stimulates and perpetuates a type 2 T helper (Th2) cell response with eventual disease chronicity and joint damage, has been suggested [4].

The majority of patients do not present with the traditional triad of arthritis, urethritis, and conjunctivitis. The disorder was formerly known as “Reiter’s syndrome,” in reference to Hans Reiter, who initially described the disease; however, this term is now best avoided because of historical and ethical considerations [5]. Up to 30% of patients experience chronic symptoms and/or complications that pose a therapeutic challenge, even though two-thirds of patients have a self-limited course and require only symptomatic and supportive treatment [1,4].

The goals of treatment in a case of ReA are mainly to provide prolonged symptomatic relief and prevent chronic complications. Early initiation and prolonged use of antimicrobial combination therapy could be an effective treatment strategy for ReA, especially in disease that develops after chlamydial infection of the genitourinary tract, an approach that has not been found to be satisfactorily effective in the post-dysenteric type of ReA [5]. In the acute phase, nonsteroidal anti-inflammatory drugs (NSAIDs), intra-articular and/or short-course systemic corticosteroids, and physical therapy constitute the first-line treatment. Disease-modifying antirheumatic drugs (DMARDs), mainly sulfasalazine and methotrexate (others include azathioprine, cyclophosphamide, and bromocriptine), are considered for patients with contraindications to NSAIDs and/or those who experience suboptimal relief despite one month of NSAID therapy.

Pending confirmation in randomized double-blind trials, anecdotal evidence suggests the effectiveness of anti-tumor necrosis factor alpha (anti-TNF-α) agents, including etanercept and infliximab, in providing substantial symptomatic relief and reduction in inflammatory markers, especially in patients who do not respond to NSAIDs and DMARDs [6]. However, TNF-α antagonists are associated with their own set of serious adverse effects, such as an increased risk of serious infections and severe glomerulonephritis, among others [7].

Unlike rheumatoid arthritis, the paradigm of “refractory disease” is not as well defined for ReA. Generally speaking, refractory disease is defined as the failure to attain predefined targets, either remission or at least a state of low disease activity with substantially controlled symptoms and disease complications. Refractory reactive arthritis (RReA) has been defined as disease persisting for more than six months, or disease that is resistant to initial therapy for acute arthritis, including NSAIDs (at least two different NSAIDs used over a total of four weeks or one month), systemic glucocorticoids (more than 7.5 mg of prednisone or its equivalent for more than three to six months), and DMARDs (no response despite administration at the maximally tolerated therapeutic dose for at least four months) [8].

For inclusion in this study, we expanded the definition of RReA by extrapolating evidence from clinical trials on refractory psoriatic arthritis (PsA) [9] to include all patients with confirmed ReA lasting for at least six months with suboptimal improvement (less than 20% improvement in swollen joint count (SJC) and tender joint count (TJC)) at 16 weeks or beyond despite at least four months (or 16 weeks) of ongoing combination therapy with at least two NSAIDs, plus oral prednisone or its equivalent at a dose of at least 7.5 mg, plus a DMARD (methotrexate and/or sulfasalazine or others administered at maximally tolerated therapeutic doses), with or without prior treatment with at least one anti-TNF drug (infliximab, adalimumab, or etanercept) at conventionally used doses and durations.

Apremilast, a phosphodiesterase-4 (PDE-4) inhibitor, has been approved for oral use by the United States Food and Drug Administration (US FDA) for the treatment of active PsA, chronic plaque psoriasis, and, more recently, Behçet’s disease-associated ulcers [10]. Additionally, it has shown promising efficacy in various inflammatory disorders of the skin, joints, and other systems. PDE4 inhibition by drugs like apremilast leads to the accumulation of intracellular cyclic adenosine monophosphate (cAMP), resulting in increased production and release of anti-inflammatory cytokines from macrophages and suppression of the inflammatory responses of macrophages, dendritic cells, Th1, Th2, and Th17 cells [11].

The overlap in pro-inflammatory and anti-inflammatory pathways in the pathophysiology of psoriasis, PsA, RA, and ReA, along with accumulating evidence supporting the efficacy of PDE-4 inhibitors in these disorders, provided the rationale for this clinical study. This study aimed to assess the efficacy and safety of oral apremilast as an adjuvant therapy administered over 40 weeks in patients with RReA. Given the rarity of RReA and the limited therapeutic options, a retrospective analysis was conducted to explore the potential role of apremilast in this setting.

## Materials and methods

Patient selection

This was a retrospective, observational pilot study conducted at a tertiary-care teaching hospital in Bihar, India. Data were collected for adult patients (age ≥18 to <65 years) of either gender with a confirmed diagnosis of ReA (as defined by the 1996 criteria of the Third International Workshop on Reactive Arthritis) [12], who met the criteria for treatment-refractory joint disease or RReA as defined previously (vide supra), from June 2024 to May 2025. Medical records of adult patients (18-65 years) with a confirmed diagnosis of reactive arthritis and normal baseline hematological, liver, and renal function tests were retrospectively reviewed. The study protocol was approved by the Institutional Ethics Committee.

Treatment exposure

All included patients had previously received oral apremilast as part of routine clinical care for RReA. Apremilast was administered at a dose of 30 mg twice daily following one week of dose titration and continued for up to 40 weeks. In this study, apremilast was used as adjuvant therapy, meaning it was added to ongoing conventional treatment regimens (NSAIDs, corticosteroids, and/or DMARDs) with the intention of enhancing therapeutic response and allowing gradual tapering of concomitant medications where clinically feasible. The concomitant pharmacological therapies (NSAIDs, oral corticosteroids, DMARDs such as weekly methotrexate, and anti-TNF biologic agents, when applicable) were initially continued but gradually titrated or tapered on a case-by-case basis according to the patient’s response after starting oral apremilast. Clinical and laboratory data recorded at baseline and at approximately eight-week intervals (8, 16, 24, 32, and 40 weeks) were extracted from patient records.

Parameters assessed and monitoring

Clinical

In addition to basic demographic and clinical parameters, patients' records were extracted at baseline and at approximately eight-weekly intervals for certain defined efficacy parameters, monitoring investigations, and adverse effects, if any (vide infra).

Evaluation of Efficacy (Outcome Measures) and Other Parameters

Treatment efficacy was evaluated based on documented clinical assessments and laboratory parameters recorded during routine follow-up visits.

Primary efficacy outcome measure (PEOM): The primary efficacy was evaluated using the ACR20 response criteria, a composite measure that includes a 20% improvement in the number of swollen and tender joints as well as a 20% improvement in three of the five criteria: erythrocyte sedimentation rate (ESR) or C-reactive protein (CRP), functional ability measure (usually the Health Assessment Questionnaire (HAQ)), patient global assessment, physician global assessment, and VAS for pain [13] 

Secondary efficacy outcome measures (SEOM): Serum C-reactive protein (CRP) levels, mean improvement from baseline to week 40, changes in pain severity (VAS), SJC, TJC, and the percentage of patients who achieved ACR50 and ACR70 responses were used to evaluate secondary efficacy outcomes. Another important SEOM was the mean change in the Health Assessment Questionnaire-Disability Index (HAQ-DI) from baseline to week 40. The HAQ-DI is a patient-reported questionnaire consisting of 20 questions across eight categories: dressing, rising, eating, walking, hygiene, reach, grip, and usual activities. An overall score ranging from 0 to 3 is calculated by averaging the scores in each category, which range from 0 (no difficulty) to 3 (unable to do) [14]. The percentage of patients achieving a minimal clinically important difference (MCID) in HAQ-DI of ≥0.35 was also evaluated.

Additionally, any treatment-related adverse events, including drug intolerance (especially nausea and diarrhoea in the first two to three follow-up visits), whether reported by the patients or observed/detected by the physician, were extracted from the patient records.

Investigations

Baseline investigations (0 week), including complete blood count (CBC), liver function test (LFT), renal function test (RFT), Elisa for HIV, HBsAg, anti-HCV antibody, urinalysis, CRP, ESR, chest X-ray, and ECG, were done. At every visit, CRP was repeated. CBC, LFT, and RFT were repeated at three-monthly intervals.

Data analysis

Data were extracted from medical records and analyzed retrospectively. Statistical analysis was performed using IBM SPSS Statistics version 25 (IBM Corp., Armonk, NY). In view of the small cohort size (n=12), the results were expressed as a comparison of the mean values of efficacy measures (with standard deviation (SD)) of SJC and TJC against the mean values at baseline of SJC and TJC, and analyzed using the Friedman test for repeated measures at eight-weekly intervals for up to 40 weeks. Categorical outcomes (ACR20/50/70 responses and HAQ-DI MCID achievement) were expressed as frequencies and percentages and were analyzed using Cochran’s Q test.

## Results

Twelve patients met the inclusion criteria described above and were assigned apremilast 30 mg twice daily for 40 weeks. All patients completed the treatment protocol and were available for analysis on all follow-up visits. At the time of inclusion, the patients' average age was 26.91 years (range: 19-36 years). They were all male. The key baseline demographic and clinical information for the 12 patients is listed in Table 1, together with information about previous and ongoing medication therapy that was continued with apremilast. Of the 12 included patients, 10 (83.33%) achieved an ACR20 response at week 40. Similarly, eight (66.67%) and five (41.67%) patients showed sustained ACR50 and ACR70 responses at 40 weeks with continued treatment, which were statistically significant (Cochran’s Q test, p<0.05). The baseline mean values of four SEOMs of all 12 patients showed improvement (reduction) over 40 weeks, including patient-reported VAS for joint pain (72.6 ±7.17 to 33.6 ±1.79); SJC (2.27 ±0.88 to 0.18 ±0.23); TJC (3.64 ±0.83 to 0.27 ±0.26); and serum CRP levels (70.91 ±26.06 to 5.36 ±2.67).

**Table 1:** Important baseline clinical information and details of ongoing pharmacological therapy* among the included patients (N=12) ^*^Before and during oral apremilast 30 mg twice-daily therapy NSAIDs: non-steroidal anti-inflammatory drugs; DMARD: disease-modifying antirheumatic drug; TNF: tumor necrosis factor

Patient	Age in years/sex	Disease duration, months	No. of different NSAIDs	Average daily dose of oral corticosteroid (prednisolone equivalent/mg per day) given over the last 6 months	DMARD/ delivery/mg	Anti-TNF agent/dose
Patient 1	34/M	6	2	O/60	MTX/O/20	-
Patient 2	22/M	12	2	O/20	MTX/O/15	Adalimumab/SC/80 MG-1st dose, then 40 MG fortnightly, 8 doses
Patient 3	19/M	9	3	O/40	MTX/O/20	Infliximab: 3 doses
Patient 4	22/M	7	2	O/25	MTX/O/10	-
Patient 5	24/M	6	3	O/40	MTX/O/15	-
Patient 6	32/M	8	3	O/50	MTX/O/20	-
Patient 7	28/M	14	3	O/25	MTX/O/15 SSZ/2000	-
Patient 8	26/M	7	2	O/30	MTX/O/15	-
Patient 9	36/M	16	2	O/20	MTX/O/20	Adalimumab/SC/80 MG-1st dose, then 40 MG fortnightly, 6 doses
Patient 10	24/M	10	3	O/30	MTX/O/20	-
Patient 11	30/M	6	3	O/40	MTX/O/15	-
Patient 12	26/M	8	2	O/40	MTX/O/15	-

At week 40, the mean SJC and TJC had improved by 79.3% and 68.8%, respectively. Additionally, there were statistically significant decreases in SJC and TJC over time (Friedman test; p<0.001) (Table 2). Patients who remained on apremilast 30 mg twice daily showed continued improvements in their physical performance through week 40, as indicated by the mean change in HAQ-DI and the fact that eight (66.67%) patients achieved a HAQ-DI MCID ≥0.35 (p<0.05).

**Table 2:** The trend of change in the primary efficacy outcome measure and secondary efficacy outcome measures evaluated over 40 weeks of apremilast treatment* ^*^[13]. ^†^Analyzed using Cochran’s Q test for repeated categorical measures. ^‡^Analyzed using the Friedman test for repeated continuous measures Use of the ACR20/50/70 response criteria is permitted for non-commercial academic purposes with attribution. ©American College of Rheumatology SJC: swollen joint count; TJC: tender joint count; HAQ-DI: Health Assessment Questionnaire-Disability Index; MCID: minimal clinically important difference

Primary and secondary efficacy outcome measures	8 weeks	16 weeks	24 weeks	32 weeks	40 weeks	Overall p*-*value
	n (%), N=12					
ACR20	2 (16.67%)	4 (33.33%)	7 (58.33%)	9 (75%)	10 (83.33%)	<0.05^†^
ACR50	1 (8.33%)	3 (25%)	5 (41.67%)	6 (50%)	8 (66.67%)	<0.05^†^
ACR70	0 (0)	2 (16.67%)	3 (25%)	5 (41.67%)	5 (41.67%)	<0.05^†^
SJC (mean percentage change reduction)	-2.4%	-12.8%	-28.6%	-56.2%	-79.3%	<0.001^‡^
TJC (mean percentage change, reduction)	-1.2%	-9.6%	-23.8%	-49.4%	-68.8%	<0.001^‡^
HAQ-DI MCID ≥0.35 (percentage change)	8.3%	33.33%	41.5%	58.3%	66.67%	<0.05^†^

No severe adverse effects or serious infections occurred in any of the patients. Most patients (n=7, 58.33%) were able to discontinue corticosteroids within a median of 12 weeks (range: 02-28 weeks) and NSAIDS (n=5, 41.66%) within a median of 16 weeks (range: 04-36 weeks) after beginning apremilast treatment. Apremilast demonstrated an acceptable safety profile. Nausea (n=5, 41.66%), headache (n=4, 33.33%), diarrhea (n=3, 25.00%), and dizziness (n=3, 25.00%) were the most common reported AEs within two weeks of apremilast treatment initiation. After two weeks, the most common AEs were nausea and headache (n=2, 16.66%). Most AEs were mild or moderate in severity. Only one patient discontinued apremilast due to a severe headache. Apremilast not only improves rheumatic symptoms but also has beneficial effects on extra-articular manifestations in refractory ReA.

## Discussion

Management of ReA, particularly in patients with refractory disease who do not respond to conventional treatments such as corticosteroids, antibiotics, DMARDs (especially methotrexate), and NSAIDs, remains challenging. In addition, there is no standardized treatment approach for those with refractory forms of ReA. Generally, the acute phase is managed with NSAIDs and corticosteroids, and the chronic phase is treated with disease-modifying anti-rheumatic drugs. However, there is no validated strategy for patients who fail to respond to these conventional drugs. Therefore, apremilast, a PDE-4 inhibitor, was evaluated, and its response was studied in 12 patients with refractory ReA.

Apremilast, a PDE-4 inhibitor, prevents the degradation of cAMP and thereby increases intracellular cAMP levels, interrupting the inflammatory cascade by reducing pro-inflammatory cytokines such as TNF-α, IL-23, and interferon-γ, and increasing anti-inflammatory mediators (e.g., IL-10) at an early stage, unlike expensive biologics that target a single pro-inflammatory marker (e.g., TNF-α) [15]. In 2014, apremilast was approved by the US FDA for the management of active PsA in adults and for moderate to severe plaque psoriasis in patients who are candidates for phototherapy or systemic therapy [16]. In addition to improvements in joint-related outcomes, a clinically meaningful corticosteroid-sparing effect was observed in several patients, with more than half able to discontinue corticosteroids during follow-up. This finding is particularly relevant in refractory ReA, where prolonged corticosteroid therapy is often associated with significant adverse effects.

In various earlier studies on the safety profile of apremilast, the drug was found to be safe, with generally well-tolerated mild to moderate adverse effects, the most common being gastrointestinal effects such as nausea, vomiting, and diarrhea [17]. Other less common adverse effects include headache, upper respiratory tract infections, weight loss, depression, and suicidal ideation. Apremilast did not show any effects on the hematological, hepatic, or renal systems.

Limitations

This study is limited by its retrospective design, small sample size, lack of a control group, and potential documentation bias. The study findings represent preliminary observational evidence of efficacy, but causal relationships cannot be definitively established.

## Conclusions

The recurrence of skin lesions and joint pain has been a major problem in the treatment of ReA. Various modalities available, including NSAIDs, corticosteroids, or DMARDs, have not been very promising in preventing recurrence and may be associated with serious adverse effects with prolonged use. It is established that the concentration of TNF-α is high in the serum and joints of patients with persistent ReA. Therefore, in this retrospective analysis, apremilast appeared to be a safe and effective adjuvant option in patients with refractory ReA and was found to be beneficial as it not only improved rheumatic symptoms but also showed beneficial effects on extra-articular manifestations and demonstrated a corticosteroid-sparing effect. Larger prospective randomized controlled trials are required to confirm the efficacy and long-term safety of apremilast in this setting. Thus, apremilast can be considered as an adjuvant in the treatment of ReA.

## Appendices

Rheumatoid Arthritis (RA) Assessment Form

For ACR 20 / 50 / 70 Response Evaluation

Study Information

Study Title: _________________________________

Study ID / Patient ID: _______________________

Visit: ☐ Baseline ☐ Follow-up (Week ___)

Date of Assessment: ____ / ____ / ______

Assessor (Clinician): _____________________

Section A: Tender Joint Count (68 joints) (Table 3).

**Table 3:** Section A: tender joint count (68 joints) (Clinician-assessed: mark 1 = tender, 0 = not tender)

Joint Area	R	L	Joint Area	R	L
TMJ	☐	☐	Shoulder	☐	☐
Elbow	☐	☐	Wrist	☐	☐
MCP 1	☐	☐	MCP 2	☐	☐
MCP 3	☐	☐	MCP 4	☐	☐
MCP 5	☐	☐	PIP 1	☐	☐
PIP 2	☐	☐	PIP 3	☐	☐
PIP 4	☐	☐	PIP 5	☐	☐
Hip	☐	☐	Knee	☐	☐
Ankle	☐	☐	MTP 1	☐	☐
MTP 2	☐	☐	MTP 3	☐	☐
MTP 4	☐	☐	MTP 5	☐	☐

Total Tender Joint Count (0-68): ______

Section B: Swollen Joint Count (66 Joints)

(Clinician-assessed: mark 1 = swollen, 0 = not swollen)

(Same joint list as above, excluding hips)

Total Swollen Joint Count (0-66): ______

Section C: Patient-Reported Outcomes

1. Patient Global Assessment of Disease Activity

Considering all the ways your arthritis has affected you in the past week, how active has your disease been?

VAS (0-100 mm):

0 (No activity) ───────────────────────────── 100 (Extremely active)

Score: ______ /100

2. Patient Pain Assessment

How much pain have you had because of your arthritis during the past week?

VAS (0-100 mm):

0 (No pain) ───────────────────────────── 100 (Worst possible pain)

Score: ______ /100

Section D: Physician Global Assessment

Physician’s overall assessment of the patient’s disease activity.

VAS (0-100 mm):

0 (No activity) ───────────────────────────── 100 (Extremely active)

Score: ______ /100

Section E: functional status (HAQ - Short Form) (Table 4)

**Table 4:** Section E: functional status (HAQ - Short Form) (Patient-reported; circle one response for each activity)

Activity	No difficulty (0)	Some difficulty (1)	Much difficulty (2)	Unable (3)
Dressing	☐	☐	☐	☐
Arising	☐	☐	☐	☐
Eating	☐	☐	☐	☐
Walking	☐	☐	☐	☐
Hygiene	☐	☐	☐	☐
Reach	☐	☐	☐	☐
Grip	☐	☐	☐	☐
Activities	☐	☐	☐	☐

HAQ Disability Index Score: ______

Section F: Acute Phase Reactant

☐ ESR: ______ mm/hr

☐ CRP: ______ mg/L

(Date of test: ____ / ____ / ______)

Section G: ACR response evaluation (for research use) (Table 5)

**Table 5:** Section G: ACR response evaluation (for research use) ACR Response Achieved: ☐ ACR20 ☐ ACR50 ☐ ACR70 ☐ No response

Measure	Baseline	Follow-up	% Improvement
Tender joint count			
Swollen joint count			
Patient global			
Patient pain			
Physician global			
HAQ			
ESR/CRP			

1. Citation

[13]
